# Volatile Organic Compound Profiles From Wheat Diseases Are Pathogen-Specific and Can Be Exploited for Disease Classification

**DOI:** 10.3389/fmicb.2021.803352

**Published:** 2022-01-07

**Authors:** Andrea Ficke, Belachew Asalf, Hans Ragnar Norli

**Affiliations:** Division of Biotechnology and Plant Health, Norwegian Institute of Bioeconomy Research (NIBIO), Ås, Norway

**Keywords:** wheat, disease, head space (HS)-GC/MS, volatiles, fungi, sensor, identificatication, classification

## Abstract

Plants and fungi emit volatile organic compounds (VOCs) that are either constitutively produced or are produced in response to changes in their physico-chemical status. We hypothesized that these chemical signals could be utilized as diagnostic tools for plant diseases. VOCs from several common wheat pathogens in pure culture (*Fusarium graminearum*, *Fusarium culmorum*, *Fusarium avenaceum*, *Fusarium poae*, and *Parastagonospora nodorum*) were collected and compared among isolates of the same fungus, between pathogens from different species, and between pathogens causing different disease groups [Fusarium head blight (FHB) and Septoria nodorum blotch (SNB)]. In addition, we inoculated two wheat varieties with either *F. graminearum* or *P. nodorum*, while one variety was also inoculated with *Blumeria graminis* f.sp. *tritici* (powdery mildew, PM). VOCs were collected 7, 14, and 21 days after inoculation. Each fungal species in pure culture emitted a different VOC blend, and each isolate could be classified into its respective disease group based on VOCs with an accuracy of 71.4 and 84.2% for FHB and SNB, respectively. When all collection times were combined, the classification of the tested diseases was correct in 84 and 86% of all cases evaluated. Germacrene D and sativene, which were associated with FHB infection, and mellein and heptadecanone, which were associated with SNB infection, were consistently emitted by both wheat varieties. Wheat plants infected with PM emitted significant amounts of 1-octen-3-ol and 3,5,5-trimethyl-2-hexene. Our study suggests that VOC blends could be used to classify wheat diseases. This is the first step toward a real-time disease detection in the field based on chemical signatures of wheat diseases.

## Introduction

As part of their metabolism, plants and fungi release diverse volatile organic compounds (VOCs) into the air. At the global level, plants alone account for 67% of the VOC emissions present in the atmosphere (Guenther, 1997; Sankaran et al., 2010). Plant VOCs are emitted in response to biotic and abiotic stresses and play an important role in defense signaling and communication between plants (Brilli et al., 2019). They can serve as priming agents to enhance resistance to both herbivores (Kim and Felton, 2013) and plant pathogens (Ameye et al., 2015) and can serve as important stimuli in the stress memory of plants. Plant VOCs can affect hormone levels and are implicated in regulating the senescence processes of competitive plants, such as weeds [reviewed in Arimura et al. (2010)]. Rhythmic release of some flower volatiles appears to be under circadian control (Fenske and Imaizumi, 2016), and the release of VOCs from oak branches fluctuated not only with light and temperature but also with the season and time of day (Pio et al., 2005). About 250 fungal-specific VOCs have been shown to have characteristic odors (Morath et al., 2012). The rate of emission of VOCs from plants and fungi varies depending on the physico-chemical and biological factors and the nature of the interaction between the pathogen and the plant (Sankaran et al., 2010; Morath et al., 2012). However, many VOCs emitted from plants in response to biotic stress factors are non-specific, as different pathogens can cause similar tissue damage and elicit the same chemical response (Jansen et al., 2011). VOC emissions have been used in pest management strategies such as pheromone-based insect control (Witzgall et al., 2010) and plant volatile-based attract-and-kill management strategies (Gregg et al., 2018). Fungal VOCs have been used as attractants and deterrents to insects and other invertebrates (Morath et al., 2012) and as resistance inducers (Naznin et al., 2013, 2014), but not as biomarkers for disease detection in the field.

Crop plants are known to emit disease-specific VOCs after pathogen infection (Shulaev et al., 1997; Cardoza et al., 2002; Algarra et al., 2015). A recent study from Thompson et al. (2021) found that Rhododendron plants infected with the oomycete *Phytophthora ramorum* had a distinctly different VOC profile than Rhododendron plants inoculated with *Phytophthora cactorum*, *Rhizoctonia solani*, and non- or mock-inoculated plants both in the growth chamber and in the field. These disease-specific VOCs could supply us with information about the pathogen’s identity and site of attack (Jansen et al., 2011) and help us to monitor diseases in real time for site-specific control.

The choice of method for collection of VOCs depends on the dynamic, type, and amounts of VOCs expected to be produced. Dynamic headspace sampling of VOCs is a frequently used method for plant VOC sampling and is characterized by a continuous stream of filtered air within a sampling container made of mostly inert, non-bleeding material, such as glass (Stewart-Jones and Poppy, 2006; Tholl et al., 2006). VOCs are captured and enriched on a separate adsorbent material that is later eluted with an organic solvent or desorbed thermally (Tholl et al., 2006). Abel et al. (2009) showed that VOC recovery rates of different target compounds could vary strongly, even if the same collection and elution method was used. Practical application of VOC detection demands portable and highly sensitive sensors. Recent advancement in this field led to the development of miniature gas-chromatography-based samplers and detector, which would facilitate the exploitation of VOCs for disease monitoring (Tholl et al., 2021).

Common wheat (*Triticum aestivum* L.) is one of the most important staple food crops in the world (Food and Agriculture Organization [FAO], 2019) and subject to several yield-reducing diseases, including Fusarium head blight (FHB), Septoria nodorum blotch (SNB), and powdery mildew (PM; Bockus et al., 2010). The potential yield loss due to pathogens was estimated to be 16% annually at the global level, increasing with production intensity (Oerke, 2006). Despite the increased use of pesticides over the last 40 years, crop losses have not decreased significantly (Oerke, 2006). This underlines the need for a more effective fungicide application. Broad-field application of fungicides, as currently practiced, applies fungicides to healthy and diseased plants within the field. Fungicides should be applied where disease is actually present. The current practice of spraying fungicides indiscriminately over the whole field leads to redundant use on areas not affected by diseases. Mapping disease hotspots in the field would enable us to apply fungicide in a site-specific manner, avoiding redundant fungicide use on healthy plants. Accordingly, the application of fungicides guided by pathogen-specific VOCs could significantly reduce the amounts of active compounds needed for disease control in the field (Gebbers and Adamchuk, 2010).

The objective of this manuscript was to determine if VOC profiles are specific for different wheat pathogens and the diseases they are causing in wheat. Accordingly, we addressed these specific hypotheses: (i) Fungal isolates causing diseases in wheat have their own characteristic VOC or VOC blend produced in pure culture. (ii) Plant pathogenic fungal isolates can be grouped into their genera/disease group based on their VOC profiles. (iii) Pathogen VOCs produced in pure culture will also be produced in plants infected with these pathogens. (iv) Infected plants will emit VOC profiles that are specific to a pathogen, allowing correct classification of the different diseases, even when different wheat varieties are used. (v) The release of pathogen-specific VOCs correlates with disease index over time.

## Materials and Methods

### Fungal and Plant Experiments

#### Fungal Maintenance and Cultivation

We used the following fungal species and isolates from the NIBIO collection of fungal cereal pathogens in the fungal VOC collection experiment: *Parastagonospora nodorum* isolates 202586, 201254, 201252, and 201253; *Fusarium culmorum* isolate 202588; *Fusarium avenaceum* isolate 202587; *Fusarium poae* isolate 202589; and *Fusarium graminearum* isolates 201569, KISH140/80, and KISH61/07. All isolates were stored at −80°C before use. Pathogens were sub-cultured and placed on potato dextrose agar (PDA, Difco™, Becton, Dickinson and Company, Le Pont de Claix, France) filled in 5-cm Petri dishes and grown for 6–10 days at 20°C and 12-h light/dark periods. Each replicate contained four 5-cm Petri dishes. The diameter of the fungal growth was determined by measuring two lines perpendicular to each other across the Petri dish from one end to the other end of the colony and taking the average of these two measurements.

#### Inoculum Preparation

*Parastagonospora nodorum* (isolate 201254) and *F. graminearum* (isolate 201569) were grown on PDA in 9-cm Petri dishes placed under near UV light, at 12-h light/dark periods and 20–25°C for 7–10 days to induce sporulation. Spore suspensions were prepared by flooding the sporulating colonies on PDA plates with sterile distilled water (0.1% Tween 20, Sigma-Aldrich, Steinheim, Germany). The suspensions were adjusted to 1 × 10^6^ spores/ml and filled into handheld pump sprayers for application referred to in the methods of Engle et al. (2003) and Lin et al. (2020). Mixed-field isolates of *Blumeria graminis* f.sp. *tritici* were collected from Norwegian spring wheat in 2010, propagated, and maintained on seedlings of spring wheat ‘Bjarne’ in the greenhouse over the course of the study.

#### Plant Cultivation and Fungal Inoculation

Spring wheat varieties ‘Bjarne’ and ‘Zebra’ were sown in 11-cm plastic pots (OS Plastic A/S Denmark) filled with the substrate perlite (grade 2, LOG AS, Oslo, Norway). The number of seedlings was thinned down to four to five plants per pot. Plants were grown in the greenhouse (15°C/18°C night/day) under 8-h dark/16-h light conditions. The plants were regularly fertilized with a balanced nutrient solution and watered. Three pots of each wheat variety were inoculated with an aqueous suspension of *P. nodorum* conidia (10^6^ spores/ml) at flag leaf stage (BBCH 47–49). Another set of three pots per variety was inoculated with an aqueous *F. graminearum* solution (10^6^ spores/ml) at flowering time (BBCH 60–65). The control plants (three pots with four to five plants per variety per inoculation time) were only sprayed with distilled water plus Tween 20 (0.1%). All plants were covered with clear plastic bags for 48 h after inoculation and kept under the same greenhouse conditions as described before. As ‘Zebra’ appeared not to be susceptible to our PM field isolate, we inoculated three pots with four to five plants of the susceptible variety ‘Bjarne’ with *B. graminis* f.sp. *tritici* by dusting conidia from young sporulating colonies onto the plants at the three-leaf stage (BBCH 13) using a fine artist’s paintbrush (Asalf et al., 2014). For powdery mildew, we used dry inoculum instead of an aqueous conidia suspension, following the method of Asalf et al. (2021). We reduced contamination of the healthy control plants by constructing a closed “mini-greenhouse.” The mini-greenhouse was constructed from a 1.5-L clear plastic bottle with its bottom removed carefully and placed over the plants in each pot. The bottle top was covered with paper tissue to allow air exchange, while preventing any cross contamination with conidia between the inoculated and control plants.

One pot with inoculated plants and one control pot per variety were chosen 7, 14, and 21 days post inoculation (dpi) for VOC collection from ‘Bjarne’ infected with *P. nodorum*, *F. graminearum*, and *B. graminis* f.sp. *tritici*. From ‘Zebra,’ we sampled plants inoculated with *P. nodorum* and *F. graminearum* 7, 14, and 21 dpi. We repeated the VOC collections for each pathogen on each of the two varieties two to four times. The 7-day sampling time was chosen to confirm successful inoculation and symptom development of all three diseases before VOC collection. The subsequent sampling times of 14 and 21 days allowed us to follow both the increase in disease index and changes in VOC production over time.

#### Disease Assessments and Plant Biomass Determination

Disease severity on leaves and stems was visually estimated as percent of plant surface area covered with brown necrotic lesions for SNB or with white mycelium for PM (Zadoks and Schein, 1979). We assessed the overall percentage of infected area from all plants in the pot. The severity of SNB and PM was determined by estimating the percent of leaf and stem area covered with light brown lesions and white powdery mycelium, respectively. The severity of FHB was determined by estimating the percentage of bleached kernels in each head. After disease assessment and VOC collection, all the above-soil parts of the plants were cut off and weighed to determine the fresh weight. The average disease severity was multiplied with the fresh weight of the plant material per pot to determine the disease index (DI) for each disease. This index was developed and used in our study to allow a more comparable disease measurement between plants at different developmental stages following the recommendations by Zadoks and Schein (1979) to define the scale in order to put disease severity into relevant context.

#### Collection of Volatile Organic Compounds From Pure Fungal Cultures

**FIGURE 1:**
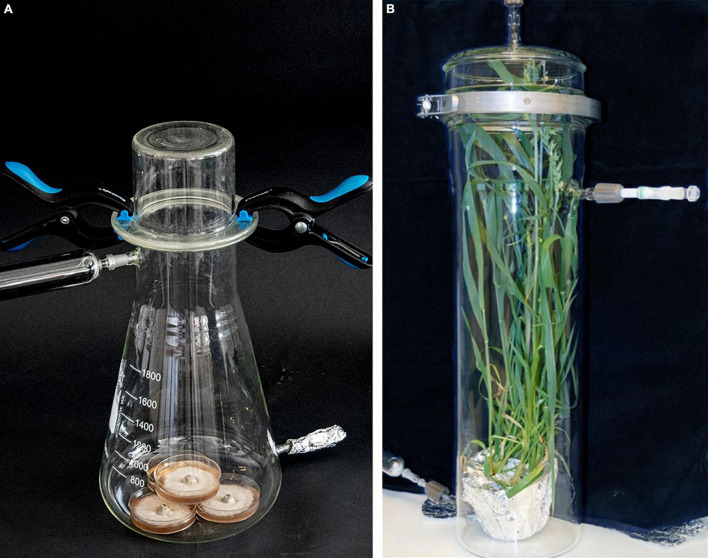
Collection of volatile organic compounds from one fungal isolate on four potato dextrose agar (PDA) plates (5-cm diameter) **(A)** and from whole wheat plants in an airtight glass container **(B)**. Coal-filtered air is pushed through the glass chamber across the fungal mycelium or the plant surface at 220–240 ml/min, and volatile organic compounds contained in the air flow are collected on Super-Q filter at the other side of the container.

Four 5-cm Petri dishes filled with PDA and covered with a specific fungal isolate, 7–10 days old, were placed like a pyramid on top of each other inside a ca. 2.5-L airtight glass container (Figure 1A). PDA plates without fungal growth were used as control. Our experimental set up allowed us to collect VOCs from four different samples at the same time in parallel. We included control samples at each sampling event. We had a total of 50 VOC collections, of which 33 were from fungi (see Table 1, i.e., sum of replicates) and 17 were control. The glass container was equipped with one inlet for charcoal-filtered air and one outlet containing a Super-Q filter (35 mg of 80/100 mesh; Alltech, Deerfield, IL, United States). Charcoal-filtered air was flowing inside the container at 220–240 ml/min for 24 h at 18 ± 2°C to collect VOCs from the headspace. The Super-Q filters were covered by aluminum foil to reduce the effect of light on the VOC during the sampling period. The Super-Q filters were cleaned by rinsing sequentially with 6 ml hexane (Sigma-Aldrich, Steinheim, Germany), 6 ml methanol (Sigma-Aldrich, Steinheim, Germany), and 6 ml hexane each time before they were used for VOC collection. The volatile compounds were desorbed from the absorbent by eluting the filter with 0.3 ml hexane, before adding 500 ng heptyl acetate (supplied from SAFC with a purity of >98%) as internal standard to the elutant. The samples were sealed in glass vials and stored at −80°C until further analyses.

**TABLE 1:** Number of replications and average sum of colony area with standard error for each fungal isolate grown on four 5-cm Petri plates at time of volatile organic compound collection (6–10 days after transfer).

Fungal species	Isolate number	Number of replicates (*n*)^b^	Sum of colony area (cm^2^) per replicate^c^
*Parastagonospora nodorum*	202586	3	32.57^BC^
*Parastagonospora nodorum*	201254^a^	4	25.73^C^
*Parastagonospora nodorum*	201252	4	29.77C
*Parastagonospora nodorum*	201253	4	43.98^B^
*Fusarium culmorum*	202588	3	78.50^A^
*Fusarium avenaceum*	202587	3	78.50^A^
*Fusarium poae*	202589	3	78.50^A^
*Fusarium graminearum*	201569^a^	3	44.00^B^
*Fusarium graminearum*	KISH140/80	3	78.50^A^
*Fusarium graminearum*	KISH61/07	3	78.50^A^

*^a^These isolates were also used in the plant inoculation.*

*^b^A replicate consisted of four Petri plates at each collection time.*

*^c^Different letters in this column indicate significant differences based on Tukey’s test with 5% error rate.*

All the glass materials used for VOC collection were washed, rinsed by spraying 70% ethanol, and then heated to 300°C for 8 h before they were reused for VOC collection. We collected VOCs from three different isolates and their control (non-inoculated PDA plates) at the same time.

#### Collection of Volatile Organic Compounds From Wheat Plants

The perlite in each pot with wheat plants was covered with aluminum foil to reduce any VOCs emitted from the growth medium and placed into glass chambers of varying sizes, adjusted to the height of the plants. Plants were placed inside the glass chambers, and the chambers were closed airtight with a ground glass fitting (Figure 1B). Depending on the disease investigated and time after inoculation, the plants were at different developmental stages when placed into the container, and the age of the plant varied somewhat with light conditions and temperature fluctuations in the green house. Wheat plants inoculated with PM at GS 13 and sampled 7, 14, and 21 dpi were approximately 3, 4, and 5 weeks old, respectively. Wheat plants inoculated with SNB at GS 47–49 were approximately 7, 8, and 9 weeks old, respectively, while wheat plants inoculated with FHB at GS 65 were approximately 9, 10, and 11 weeks old, respectively. Charcoal-filtered clean air was pushed through the chamber at a rate of 220–240 ml/min. A Super-Q filter (adsorbent tube) was connected to the chambers’ exhaust to collect VOCs from the plant’s headspace as described above for 24 h at 20 ± 2°C. The Super-Q filters were covered by aluminum foil during the sampling period as described above. The VOCs were adsorbed, 500 ng of the heptyl acetate standard was added, and the glass vials were stored as described above. We collected VOCs from four glass containers (each containing one pot with plants) at the same time, which included ‘Bjarne’ and ‘Zebra’ inoculated with the pathogen and their non-inoculated controls.

### Chemical Analysis and Compound Identification

#### Gas Chromatography and Mass Spectrometry

Instrument settings for volatile analysis, including gas chromatography (GC) column type and temperature program together with mass spectrometry (MS) parameters, are described by Dalen et al. (2015).

#### Peak Selection and Determination of Relative Amount of Volatile Organic Compounds

The Deconvolution Reporting Software (DRS—version A.02.00, Agilent Technologies) in combination with the Automated Mass Spectral Deconvolution and Identification System (AMDIS) database^1^ was used for identification of volatile compounds. Details regarding DRS and AMDIS settings are described by Steen et al. (2019). Peaks present in the chromatogram but not identified by the DRS were manually interpreted through the NIST08-database. For reliable identification, a match factor ≥70 was employed (Stein, 1999). Additionally, MetAlign, version 04101 settings as in Steen et al. (2019), was used to investigate if compounds missed by AMDIS or NIST would appear by differential analysis of data sets comparing inoculated and non-inoculated PDA Petri dishes and inoculated and control plants (Tikunov et al., 2005). Compounds of interest were selected if they were consistently detected in the fungal samples or inoculated plants. Relative amounts of microgram heptyl acetate equivalents of identified compounds were calculated by dividing the peak area (using the area from the total ion chromatogram) by the area of the internal standard heptyl acetate. The VOCs octanal, 1-octen-3-ol, sativene, 1-octanol, germacrene D, and mellein were verified by comparison with synthetic standards. The compound mellein was provided by Yngve H. Stenstrøm (Norwegian University of Life Sciences, Ås, Norway). All other standards were supplied by Sigma-Aldrich, Fluka, Chiron AS, Supleco, and SAFC with a purity ranging from 95 to 99%.

### Statistical Analysis

#### Fungal Growth on Potato Dextrose Agar

We calculated the average sum of the colony areas for each repeated experiment. One experiment consisted of collecting VOCs from three isolates (four 5-cm Petri dishes) and one control (four 5-cm Petri dish only filled with PDA) in parallel. The experiment was repeated three to four times. We compared fungal growth using Tukey’s test with an error rate of 5% assuming equal variance.

#### Disease Severity, Fresh Biomass, and Disease Index

The disease severity, biomass, and disease index data taken from inoculated wheat plants were normally distributed, and we calculated the means of the disease severity 7, 14, and 21 dpi. We compared disease severity, fresh biomass, and disease index between different collection times (7, 14, and 21 dpi) for each variety–pathogen combination using Tukey’s test for pairwise comparison with an error rate of 5% assuming equal variance. There was no disease on plants not inoculated with the different pathogens, and the disease index was therefore zero during each of the collection times for the controls. Data on disease severity, biomass, and disease index were therefore not analyzed statistically for non-inoculated plants. One experiment consisted of one pot inoculated and one pot control ‘Zebra’ plants and one pot of inoculated and one pot of control ‘Bjarne’ plants. We repeated the collection of VOCs from ‘Zebra’ and ‘Bjarne’ plants three to four times, except for the collection of VOCs from ‘Bjarne’ plants at 14 dpi with PM, which we repeated two times.

#### Determining Which Volatile Organic Compounds Were Produced by Fungal Isolates *in vitro*

The median amounts for each of the selected VOCs for all replicates were calculated for each isolate. The number of replicates was three for all *F. graminearum* isolates; four for *F. avenaceum*, *F. culmorum*, and *F. poae* isolates; and five for all *P. nodorum* isolates, except for *P. nodorum* isolate number 201254, where we repeated the collection four times. We included the control (PDA only) during each collection event (17 control samples in total). The data for the different VOCs collected from inoculated PDA plates were not normally distributed, as there were many zeros in the data set. Transformation of the data did not normalize the data, and we decided to use a non-parametric Kruskal–Wallis test to determine if the median amount of each collected compound differed significantly between isolates and control plates. *P*-values were adjusted for ties, and the significance level was set to *P* < 0.05 (Table 2).

**TABLE 2:** Volatile organic compounds in headspace collections from fungal isolates on potato dextrose agar (PDA).

DB-WAX	RI	Compound (Identified by AMDIS)	CAS Nr	*P* value	Amount of compounds trapped in 24 h are presented as microgram heptyl acetate equivalents									
					Fusarium head blight^c^						Septoria nodorum blotch^c^			
					*Fusarium graminearum*			*Fusarium avenaceum*	*Fusarium culmorum*	*Fusarium poae*	*Parastagonospora nodorum*			
							
RT					201569 (*n* = 3)^b^	KISH 140/80 (*n* = 3)^b^	KISH 61/07 (*n* = 3)^b^	203587 (*n* = 4)^b^	202588 (*n* = 4)^b^	202589 (*n* = 4)^b^	201254 (*n* = 4)^b^	201252 (*n* = 5)^b^	201253 (*n* = 5)^b^	202586 (*n* = 5)^b^
9.05	1,285	**Octanal^a^**	124-13-0	0.010	0	0	0	0.005	0	0.09	0	0	0	0
11.09	1,390	2-Non-anone	124-19-6	0.042	0.088	0.052	0.052	0.05	0.056	0.05	0.222	0.066	0.043	0.064
12.22	1,451	**1-Octen-3-ol^a^**	3391-86-4	<0.001	0.06	0.425	0.303	0.036	0.214	0.029	0.888	0.616	0.944	2.205
12.84	1,482	3,5,5-Trimethyl-2-hexene	26456-76-8	<0.001	0.108	0.094	0.042	0.004	0.013	0.024	0.265	0.01	0.338	0.237
13.58	1,523	**Sativene^a^**	3650-28-0	0	0	0	0	0	0	0	0	0	0	0
14.17	1,559	**1-Octanol^a^**	111-87-5	<0.001	0	0	0	0	0	0.014	0	0	0	0
14.26	1,564	Alpha-cedrene	469-61-4	0.002	0	0		0	0	0.033	0.057	0	0	0.054
16.64	1,702	**Germacrene D^a^**	23986-74-5	<0.001	0.026	0.042	0	0	0.02	0.475	0.162	0	0	0.429
17.44	1,748	α-Patcholene	560-32-7	<0.001	0.03	0.03	0.047	0	0	0.357	0.166	0.011	0	0.288
17.56	1,757	2-(1-Cyclopent-1-enyl-methylethyl) cyclopentanone	NA (NIST# 62949)	<0.001	0.115	0.118	0.359	0	0	1.219	0	0	0	0.046
20.93	2,020	2-Pentadecanone	2345-28-0	0	0	0	0	0	0	0	0	0	0	0
21.35	2,070	Acoradiene	24048-44-0	<0.001	0	0	0	0	0	0.273	0	0	0	0
21.50	2,088	Dihydro-beta-ionone	17283-81-7	<0.001	0	0	0	0	0	0.526	0	0	0	0
22.42	2,232	2-Heptadecanone	2922-51-2	0	0	0	0	0	0	0	0	0	0	0
**23.75**	2,507	**Mellein^a^**	17397-85-2	<0.001	0	0	0	0	0	0	2.754	24.858	2.732	23.544

*Median is given in the table for the different compounds. If the P value was less than 0.05, at least one median was different from the rest. Gray cells indicate which median of which compounds was above zero.*

*^a^Compounds in bold were verified by comparison with synthetic standards.*

*^b^Number of replicates is given in parenthesis after isolate number.*

*^c^Amount of compounds trapped in 24 h is presented as microgram heptyl acetate equivalents from different isolates grown on PDA in four 5-cm Petri dishes. Volatile organic compounds were collected from 6- to 10-day-old cultures. Due to non-normality, the use of the non-parametric Kruskal–Wallis test was warranted. P values were adjusted for ties.*

#### Classification of Fungal Cultures Into Their Respective Isolate and Disease Group

We used a discriminant analysis based on a linear model to group the isolates according to the different VOCs produced. “Fungal isolate” (including the PDA control) was the response factor, and a subset of all VOCs produced in significant amounts by the isolates (Table 2) was the predictor [2-non-anone; 1-octen-3-ol; 3,5,5-trimethyl-2-hexene; germacrene D; α-patcholene; 2-(1-cyclopent-1-enyl-methylethyl)cyclopentanone; acoradiene; and mellein] to classify the isolates into their respective isolate groups. We omitted alpha-cedrene and dihydro-beta-iodine in the classification as these were strongly correlated with the other predictors. The fungal isolates were grouped into their respective disease groups (control, FHB, and SNB). The disease group FHB contained all *F. graminearum*, *F. avenaceum*, *F. culmorum*, and *F. poae* isolates, while the SNB group contained all *P. nodorum* isolates. Only octanal; 1-octen-3-ol; 3,5,5-trimethyl-2-hexene; germacrene D; α-patcholene; 2-(1-cyclopent-1-enyl-methylethyl); and mellein were used as predictors for “disease” as we again omitted those VOCs that correlated strongly with other predictors. Cross validation was used to test the robustness of the classification system.

#### Determining Which Volatile Organic Compounds Were Produced by Two Different Wheat Varieties Inoculated With Different Pathogens

The median amounts for each of these selected VOCs for all replicates were calculated for each pathogen–variety–time after inoculation combination. Each experiment (pathogen–variety–time after inoculation combination) was repeated three–four times, except for the collection of VOCs from ‘Bjarne’ inoculated with PM at 14 dpi. The data was not normally distributed due to many zeros in the dataset. Transformation of the data did not improve normality. Therefore, we used a non-parametric Kruskal–Wallis test to test if the median amounts of each collected compound differed significantly between the treatments (FHB, SNB, control, and PM), separately for one wheat variety (‘Bjarne’ or ‘Zebra’) at each collection time (7, 14, and 21 dpi). This gave a total of 225 tests {[2 varieties × 2 diseases (FHB and SNB) × 3 collection times × 15 compounds] + [1 variety × 1 disease (PM) × 3 collection times × 15 compounds]}. *P*-values were adjusted for ties, and the significance level was set to 0.05 (Table 3).

**TABLE 3:** Volatile organic compounds in headspace collections from potted wheat plants (‘Zebra’ and ‘Bjarne’) 7 (A), 14 (B), and 21 (C) days after inoculation with *Fusarium graminearum* or *Parastagonospora nodorum*. Mean release rates of compounds trapped over 24 h are presented as heptyl equivalents per pot. The amounts of the different compounds are given in medians. If the *P* value was less than 0.05, one treatment median per variety was significantly different from the rest (Kruskal–Wallis test, gray cells). *P* values were adjusted for ties. Only compounds that were produced in significantly higher amounts than from the control are listed in the table.

(A)										
				‘Zebra’			‘Bjarne’			
RT	RI	Compound (identified by AMDS)	Cas Nr	*P* value	*P. nodorum* (*n* = 4)^b^	*F. graminearum* (*n* = 3)^b^	*P* value	*P. nodorum* (*n* = 4)^b^	*F. graminearum* (*n* = 3)^b^	*B. graminis* (*n* = 3)^b^
12.84	1,482	3,5,5-Trimethyl-2-hexene	26456-76-8	0.14	0.006	0.016	0.035	0.006	0.013	0.032
13.58	1,523	**Sativene^a^**	3650-28-0	0.02	0	0.019	0.01	0	0.021	0
14.26	1,564	Alpha-cedrene	469-61-4	0.17	0.001	0.012	0.026	0.004	0.012	0
16.64	1,702	**Germacrene D^a^**	23986-74-5	0.012	0	0.035	0.002	0	0.043	0
17.56	1,757	2-(1-Cyclopent-1-enyl-methylethyl) cyclopentanone	NA	0.005	0	0.256	0.002	0	0.253	0
			(NIST# 62949)							
22.42	2,232	2-Heptadecanone	2922-51-2	0.039	0.045	0	0.002	0.021	0	0

**(B)**										

12.22	1,451	**1-Octen-3-ol^a^**	3391-86-4	0.06	0.029	0.117	0.036	0.022	0.066	0.312
12.84	1,482	3.5.5-Trimethyl-2-hexene	26456-76-8	0.018	0.007	0.038	0.018	0.006	0.02	0.126
13.58	1,523	**Sativene^a^**	3650-28-0	0.005	0	0.007	0.06	0	0.009	0
16.64	1,702	**Germacrene D^a^**	23986-74-5	0.013	0	0.069	0.005	0	0.037	0
17.44	1,748	α−Πατχηoλενε	560-32-7	0.005	0	0.011	0.06	0	0.012	0
17.56	1,757	2-(1-Cyclopent-1-enyl-methylethyl) cyclopentanone	NA	0.005	0	0.149	0.014	0	0.136	0
			(NIST# 62949)							
21.35	2,070	Acoradiene	24048-44-0	0.005	0	0.007	0.06	0	0.006	0
21.5	2,088	Dihydro-beta-ionone	17283-81-7	0.005	0	0.004	0.06	0	0.003	0
22.42	2,232	2-Heptadecanone	2922-51-2	0.084	0.051	0.005	0.016	0.039	0	0
23.75	2,507	**Mellein^a^**	17397-85-2	0.033	0.003	0	0.038	0.006	0	0

**(C)**										

12.22	1,451	**1-Octen-3-ol^a^**	3391-86-4	0.068	0.084	0.134	0.018	0.047	0.101	0.781
12.84	1,482	3,5,5-Trimethyl-2-hexene	26456-76-8	0.036	0.009	0.029	0.013	0.01	0.026	0.352
13.58	1,523	**Sativene^a^**	3650-28-0	0.005	0	0.014	0.046	0	0.006	0
14.26	1,564	Alpha-cedrene	469-61-4	0.049	0.006	0.013	0.022	0.005	0.014	0
16.64	1,702	**Germacrene D^a^**	23986-74-5	0.005	0	0.073	0.016	0	0.045	0
17.44	1,748	α−Πατχηoλενε	560-32-7	0.005	0	0.018	0.148	0	0.009	0
17.56	1,757	2-(1-Cyclopent-1-enyl-methylethyl) cyclopentanone	NA	0.005	0	0.238	0.065	0	0.11	0.014
			(NIST# 62949)							
20.93	2,020	2-Pentadecanone	2345-28-0	0.113	0.57	0.103	0.024	0.31	0.734	0
21.35	2,070	Acoradiene	24048-44-0	0.005	0	0.012	0.046	0	0.008	0
21.5	2,088	Dihydro-beta-ionone	17283-81-7	0.005	0	0.004	0.046	0	0.003	0
22.42	2,232	2-Heptadecanone	2922-51-2	0.116	0.014	0	0.003	0.033	0	0
23.75	2,507	**Mellein^a^**	17397-85-2	0.033	0.003	0	0.003	0.027	0	0

*^a^Compounds in bold were verified by comparison with synthetic standards.*

*^b^Number of replicates are given in parenthesis after the name of the disease.*

#### The Classification of Fungal Diseases Into Their Respective Disease Group

We used a discriminant analysis based on a linear model to group the diseases on both varieties and the control 7, 14, and 21 dpi. We only considered those compounds in the discriminant analysis that were produced in significant amounts as predictors for each variety separately (Table 3). We found that 2-(1-cyclopen-1-enyl-methylethyl)cyclopentanone, dihydro-beta-iodine, α-patcholene, and dihydro-beta-ionine were highly correlated with other VOCs at 14 and 21 dpi in the ‘Zebra’ samples, so we omitted these compounds in the discriminant analysis and only used 3,5,5-trimethyl-2-hexene; sativene; germacrene D; and 2-heptadecanone to classify the diseases 7 and 14 dpi. Germacrene D was highly correlated with the other predictors at 21 dpi, and mellein was added to the predictors instead. For classification of diseases at 7 dpi on ‘Bjarne,’ we used 1-octen-3-ol; 3,5,5-trimethyl-2-hexene; 1-octanol; sativene; alpha-cedrene; germacrene D; 2-(1-cyclopent-1-enyl-methylethyl)cyclopentanone; and 2-heptadecanone as the predictors. After 14 dpi, we classified the diseases on ‘Bjarne’ into their respective groups using 1-octen-3-ol; 3,5,5-trimethyl-2-hexene; 1-octanol; sativene; alpha-cedrene; and 2-heptadecanone. The classification of diseases on ‘Bjarne’ into their respective groups 21 dpi was based on only 1-octen-3-ol; 3,5,5-trimethyl-2-hexene; alpha-cedrene; germacrene D; mellein; and α-patcholene.

We then combined the VOC data for all sampling times for ‘Zebra’ (predictors included 3,5,5-trimethyl-2-hexene; sativene; germacrene D; mellein; and 2-heptadecanone) and did the same for ‘Bjarne’ [predictors included 1-octen-3-ol; 3,5,5-trimethyl-2-hexene; alpha-cedrene; germacrene D; 2-(1-cyclopent-1-enyl-methylethethyl)cyclopentanone; 2-pentadecanone; 2-heptadecanone; mullein; and 1-octanol] to see if the VOC blend-based classification was correctly putting the diseases into their respective groups independent of how long after infection the VOCs were collected from the two different varieties. Finally, we combined the data from all sample times and both varieties to see if the classification would work independent of the variety we used [predictors included 1-octen-3-ol; 3,5,5-trimethyl-2-hexene; sativene; germacrene D; α-patcholene; 2-(1-cyclopent-1-enyl-methylethethyl)cyclopentanone; mellein; and 2-heptadecanone]. We used cross validation to test the robustness of the classification model.

#### Correlation of Volatile Organic Compounds With Disease Index Across Wheat Varieties

We regressed the DI of SNB, FHB, and PM with VOCs produced in significant amounts after inoculation from each and then from both wheat varieties over time. Based on the Table 3, we chose to regress mellein and heptadecanone against the DI of SNB as they were consistently produced by ‘Zebra’ and/or ‘Bjarne’ in significant amounts. For FHB, we chose germacrene D and sativene as they were the VOCs produced by ‘Zebra’ and/or ‘Bjarne’ at all collection times. Both 1-octen-3-ol and 3,5,5-trimethyl-2-hexene were produced in significant amounts by ‘Bjarne’ inoculated with PM at most sampling times, and both compounds were regressed against the DI of PM. We compared the regression models for each disease–VOC relationship between the two different varieties using “variety” as a categorical predictor and “DI × variety” as the interaction term.

## Results

### Fungal Growth and Disease Severity in Plants

The average colony area grown on four Petri dishes for each isolate at the time of VOC collection is given in Table 1. Isolates of *F. culmorum*, *F. avenaceum*, and *F. poae* and isolates KISH140/80 and KISH61/07 of *F. graminearum* covered the entire area of each Petri dish (19.63 cm^2^), amounting to 78.54 cm^2^ for four Petri dishes at each collection time, while *F. graminearum* isolate 201569 covered on average 44.00 cm^2^, and the four *P. nodorum* isolates covered an area from 25.73 to 43.98 cm^2^ per collection time (Table 1).

The average disease severity, fresh biomass, and the DI on wheat variety ‘Bjarne’ and ‘Zebra’ at the time of VOC collection and 7, 14, and 21 dpi with FHB, SNB, and/or PM are given in Table 4. The average FHB severity increased from 30 to 55% and from 29 to 55% between day 7 and day 21 on ‘Zebra’ and ‘Bjarne,’ respectively. The average SNB severity on ‘Zebra’ and ‘Bjarne’ increased from 1.6 to 14% between 7 and 21 days and from 4.3 to 32%, during the same time, respectively. The average PM severity on ‘Bjarne’ almost doubled from 48% at 7 dpi to 84% at 21 dpi. There was no disease development visible on the control plants. On ‘Zebra,’ the fresh weight from the FHB experiments varied between 48.25 and 59.3 g and from the SNB experiments between 46.5 and 53.26 g. The fresh weight of ‘Bjarne’ plants used for each of the FHB experiments varied between 38.66 and 44.53 g, between 35.97 and 56.49 g for the SNB experiments, and from 4.64 to 5.71 g for seedlings in the PM experiments. Accordingly, the DI across disease, variety, and day after inoculation combinations varied considerably between 1,068 and 3.434 for FHB and between 99 and 702 for SNB on ‘Zebra’ between 7 and 21 dpi. On ‘Bjarne,’ the DI for FHB during the same time interval varied between 1,394 and 2,237, for SNB between 134 and 1,830, and for PM between 246 and 521 (Table 4). There was no significant increase in the average disease severity, fresh biomass, or DI for FHB and SNB on ‘Zebra’ and FHB on ‘Bjarne.’ However, these parameters increased significantly with time after inoculation for SNB on ‘Bjarne.’

**TABLE 4:** Overview of experimental set up for potted plant inoculations, wheat varieties and isolates used, sampling time, average and highest disease severity, fresh biomass (g), and disease index (average severity multiplied with biomass).

	**‘Zebra’** ^a^						**‘Bjarne’** ^a^								
	***Fusarium graminearum* (201569)**			***Parastagonospora nodorum* (201254)**			***Fusarium graminearum* (201569)**			***Parastagonospora nodorum* (201254)**			***Blumeria graminis* (field isolate)**		
Days after inoculation	7 (3)	14 (3)	21 (3)	7 (4)	14 (4)	21 (4)	7 (3)	14 (3)	21 (3)	7 (4)	14 (4)	21 (4)	7 (3)	14 (2)	21 (3)
Average severity (%)	30.0 A ± 15.3	21.7 A ± 9.3	55.0 A ± 18.9	1.6 A ± 0.6	11.3 A ± 3.2	14.0 A ± 5.0	29.0 A ± 16.9	38.0 A ± 19.1	55.0 A ± 25.2	4.3 B ± 1.6	14.1 AB ± 4.5	32.0 A ± 10.2	48.1 B ± 6.9	79.5 A ± 1.6	84.2 A ± 7.9
Fresh biomass (g)	51.62 A ± 4.7	48.25 A ± 1.2	59.26 A ± 5.8	48.87 A ± 5.5	53.26 A ± 5.6	46.50 A ± 4.2	44.21 A ± 3.6	44.53 A ± 6.5	38.66 A ± 3.6	35.97 B ± 6.2	39.77 AB ± 2.1	56.49 A ± 4.5	4.64 A ± 1.8	5.91 A ± 2.0	5.71 A ± 3.4
Disease index	1,453 A ± 632	1,068 A ± 484	3,434 A ± 1,288	99 A ± 30	604 A ± 204	702 A ± 298	1,394 A ± 840	1,901 A ± 1,135	2,237 A ± 1,038	134 A ± 34	544 AB ± 151	1,830 B ± 580	246 A ± 119	464 A ± 148	521 A ± 334

*^a^Number of replicates are given in parenthesis beside the number of days after inoculation; standard error is given after the plus/minus (±) sign. Means that share a letter are not significantly different based on Tukey’s pairwise comparison of days after inoculation for each variety–pathogen combination with 95% confidence.*

### Volatile Organic Compounds From Fungal Isolates in Pure Culture

MetAlign grouping selected the following 15 compounds to be significant in distinguishing between fungal isolates on PDA and control (PDA only): octanal; 2-non-anone; 1-octen-3-ol; 3,5,5-trimethyl-2-hexene; 1-octanol; alpha-cedrene; sativene; germancrene D; α-patcholene; 2-(1-cyclopent-1-enyl-methylethyl)cyclopentanone; acoradiene; dihydro-beta-ionone; 2-pentadecanone; 2-heptadecanone; and mellein. The retention time, retention index, and CAS number [a unique number assigned by the Chemical Abstracts Service (CAS)] of the 15 compounds are presented in Table 2. The compound 1-octen-3-ol was sampled in significant amounts from all isolates tested on PDA, except from *F. avenaceum*, while 2-non-anone was sampled from all isolates except from *F. graminearum* KISH140/80 and KISH61/07. The compound 3,5,5-trimethyl-2-hexene was sampled from all isolates except from *F. avenaceum* and *F. culmorum* (Table 2).

Most isolates emitted a unique blend of VOCs (Table 2). *F. graminearum* isolate 201569 emitted 2-non-anone; 1-octen-3-ol; and 3,5,5-trimethyl-2-hexene. Both *F. graminearum* isolate 140/80 and 61/07 emitted 1-octen-3-ol; 3,5,5-trimethyl-2-hexene; and 2-(1-cyclopent-1-enyl-methylethyl)cyclopentanone, but isolate140/80 emitted germacrene D in addition to those compounds. *F. avenaceum* (isolate 203587) emitted only 2-non-anone, and *F. culmorum* (isolate 202588) emitted 2-non-anone and 1-octen-3-ol. *F. poae* (isolate 202589) emitted octanal; 2-non-anone; 1-octen-3-ol; 3,5,5-trimethyl-2-hexene; alpha-cedrene; germancrene D; α-patcholene; 2-(1-cyclopent-1-enyl-methylethyl)cyclopentanone; acoradiene; and dihydro-beta-ionone (Table 2).

All four isolates from the SNB pathogen *P. nodorum* produced 2-non-anone; 1-octen-3-ol; 3,5,5-trimethyl-2-hexene; and mellein. In addition, both *P. nodorum* isolate 201254 and isolate 202586 produced alpha-cedrene, germacrene D, and α-patcholene; isolate 201252 produced α-patcholene; and isolate 201253 produced no other additional VOCs (Table 2).

Linear discriminant analysis based on a linear model with “isolate” as the response and all compounds produced in significant amounts included as predictors [octanal; 2-non-anone; 1-octen-3-ol; 3,5,5-trimethyl-2-hexene; germacrene D; α-patcholene; 2-(1-cyclopent-1-enyl-methylethyl; acoradiene; and mellein as the predictors] showed an overall correct classification of 84.2%. Cross validation showed that 68.4% of isolates were classified correctly based on these nine VOCs chosen for the analysis. *F. graminearum* isolate 201569 and isolate KISH140/80, *F. poae*, *P. nodorum* isolate 201254, and the PDA control were classified 100% correctly into their isolate group, while *P. nodorum* isolate 201253, F. *avenaceum*, and *F. graminearum* isolate KISH61/70 were classified 80, 75, and 67% correctly (Table 5). *P. nodorum* isolate 202586, *P. nodorum* isolate 201252, and *F. culmorum* were classified 60, 50, and 50% correctly into their fungal isolate group, respectively, based on the selected volatiles (Table 5). Cross validation showed a lower level of correct classification than during the initial analysis except for F. *avenaceum* and *F. graminearum* isolates KISH140/80 and KISH61/70, which was correctly classified in 75, 100, and 67% of the cases, respectively (Table 5). Correct classification with cross validation was 25% for *F. culmorum*, 67% for *F. graminearum* isolate 201569, 75% for *F. poae*, and 88% for the PDA control plates (Table 5). *P. nodorum* isolates 202586, 201253, 201252, and 201254 were correctly classified in 60, 40, 25, and 80% of all cases, respectively, after cross validation. Then, we combined the *F. graminearum*, *F. avenaceum*, *F. culmorum*, and *F. poae* isolates into one group of Fusarium head blight pathogens and all *P. nodorum* isolates into the SNB group. After this grouping of the isolates into their disease groups, we had an overall correct classification into disease groups in 48 out of 57 cases or 84.2%, when we used “disease group” as the response and 1-octen-3-ol; 3,5,5-trimethyl-2-hexene; germacrene D; α-patcholene; 2-(1-cyclopent-1-enyl-methylethyl); and mellein as the predictors. All 17 controls were placed correctly into the control group. Six of the FHB isolates were also grouped into the control group, while 15 were grouped correctly into the FHB disease group (71.4%). Sixteen out of 19 SNB isolates were correctly placed into the SNB group, while two were placed into the FHB group and one into the control group (84.2% correct classification) (Table 6). Based on the selected compounds, cross validation showed a somewhat lower overall rate of correct classification, 77.2%, with 61.9% correct classification of FHB isolates and 73.7% correct classification of SNB isolates (Table 6).

**TABLE 5:** summary of classification for fungal isolates in pure culture based on linear discriminant analysis with each fungal isolate as the response factor and octanal; 2-non-anone; 1-octen-3-ol; 3,5,5-trimethyl-2-hexene; germacrene D; α-patcholene; 2-(1-cyclopent-1-enyl-methylethyl) cyclopentanone; acoradiene, and mellein as the predictors.

	True group										
Put into group	*F. avenaceum*	*F. culmorum*	*F.* *graminearum* (201569)	*F. graminearum* (KISH 140/80)	*F. graminearum* (KISH 61/70)	*F. poae*	PDA	*P. nodorum* (202586)	*P. nodorum* (201253)	*P. nodorum* (201252)	*P. nodorum* (201254)
*F. avenaceum*	3	2	0	0	0	0	0	0	0	0	0
*F. culmorum*	1	2	0	0	0	0	0	1	1	0	0
*F. graminearum* (201569)	0	0	3	0	0	0	0	0	0	2	0
*F. graminearum* (KISH61/80)	0	0	0	3	1	0	0	0	0	0	0
*F. graminearum* (KISH61/70)	0	0	0	0	2	0	0	0	0	0	0
*F. poae*	0	0	0	0	0	4	0	0	0	0	0
*PDA*	0	0	0	0	0	0	17	1	0	0	0
*P. nodorum* (202586)	0	0	0	0	0	0	0	3	0	0	0
*P. nodorum* (201253*)*	0	0	0	0	0	0	0	0	4	0	0
*P. nodorum* (201252)	0	0	0	0	0	0	0	0	0	2	0
*P. nodorum* (201254)	0	0	0	0	0	0	0	0	0	0	5
Total *N*	4	4	3	3	3	4	17	5	5	4	5
Correct *N*	3	2	3	3	2	4	17	3	4	2	5
Correctly classified (%)	75	50	100	100	67	100	100	60	80	50	100
Correctly classified after cross validation	75	25	67	100	67	75	88	60	40	25	80

**TABLE 6:** Summary of classification for fungal isolates grouped into diseases based on linear discriminant analysis with disease group as a response and 1-octen-3-ol; 3,5,5-trimethyl-2-hexene; germacrene D; α-patcholene; 2-(1-cyclopent-1-enyl-methylethyl)cyclopentanone; and mellein as the predictors.

Put into group	True group		
	Control	Fusarium head blight	Septoria nodorum blotch
Control	17	6	1
Fusarium head blight	0	15	2
Septoria nodorum blotch	0	0	16
Total *N*	17	21	19
Correct *N*	17	15	16
Correctly classified (%)	100	71.4	84.2
Correct after cross validation (%)	100	61.9	73.7

### Volatile Organic Compounds From Inoculated Plants

#### Identity of Volatile Organic Compounds Produced

MetAlign grouping selected the following 12 compounds to be significant in distinguishing between inoculated and control plants at 7, 14, and 21 dpi (Table 3). Each disease on ‘Zebra’ and ‘Bjarne’ had a distinct VOC profile. The production of some compounds was only observed at some, but not all, collection times. ‘Zebra’ and ‘Bjarne’ both emitted sativene, germacrene D, and 2-(1-cyclopent-1-enyl-methylethyl)cyclopentanone 7 days after inoculation with *F. graminearum*. However, while ‘Zebra’ produced 3,5,5-trimethyl-2-hexene, ‘Bjarne’ produced alpha-cedrene in addition to those compounds 7 days after inoculation (Table 3A). Both cultivars produced 2-heptadecanone 7 days after inoculation with *P. nodorum* (Table 3A). Seven days after inoculating ‘Bjarne’ with the powdery mildew pathogen *B. graminis*, the plants emitted 3,5,5-trimethyl-2-hexene.

After 14 days of inoculation with *F. graminearum*, ‘Zebra’ and ‘Bjarne’ both still produced germacrene D and 2-(1-cyclopent-1-enyl-methylethyl)cyclopentanone, while only ‘Zebra’ produced sativene. In addition, ‘Zebra’ also produced 3,5,5-trimethyl-2-hexene, α-patcholene, acoradiene, and dihydro-beta-ionone after inoculation with *F. graminearum* (Table 3B). Fourteen days after inoculation with *P. nodorum*, both varieties produced mellein, but ‘Bjarne’ also produced 2-heptadecanone in addition to that (Table 3B). ‘Bjarne’ produced 1-octen-3-ol and 3,5,5-trimethyl-2-hexene 14 dpi with *B. graminis*.

Twenty-one days after inoculating ‘Zebra’ and ‘Bjarne’ with *F. graminearum*, both varieties produced again sativene and germacrene D (Table 3C). In addition, ‘Zebra’ produced α-patcholene, 2-(1-cyclopent-1-enyl-methylethyl)cyclopentanone, acoradiene, and dihydro-beta-ionone. ‘Bjarne’ plants produced alpha-cedrene and 2-pentadecanone in addition to sativene and germacrene at 21 dpi with *F. graminearum* (Table 3C). Twenty-one days after inoculation with *P. nodorum*, ‘Zebra’ and ‘Bjarne’ both produced mellein, while ‘Bjarne’ also produced 2-heptadecanone in significant amounts (Table 3C). After 21 days, plants inoculated with *B. graminis* produced 1-octen-3-ol and 3,5,5-trimethyl-2-hexene in significant amounts (Table 3C).

#### Classification of Fungal Diseases Into Their Respective Disease Group

The discriminant analysis of the two diseases FHB and SNB on ‘Zebra’ with “disease” (“control,” “FHB,” and “SNB”) as response factor and several selected volatiles as predictors correctly classified the diseases in 79, 86, and 86% for 7, 14, and 21 dpi, respectively. All “control” cases 7 and 14 dpi were correctly classified, while one of the seven control cases was classified as SNB at 21 dpi (Table 7). Fusarium head blight on ‘Zebra’ was 100% correctly classified at 14 and 21 dpi and 67% at 7 dpi (Table 7). SNB was correctly classified in 50% of the cases at 7 and 14 dpi and in 75% at 21 dpi (Table 7). Cross validation revealed a lower rate of correct classification with only 86% correct classifications of the control cases at all collection times. At 7, 14, and 21 dpi 33, 67, and 67% of FHB cases were correctly classified, respectively, while 50, 55, and 50% of SNB cases were correctly classified at those time points using cross validation (Table 7). The discriminant analysis of the three different diseases on ‘Bjarne,’ with “disease” (“control,” “FHB,” “PM,” and “SNB”) as response factor and different volatiles as predictors, classified 85%, 94%, and 95% of the cases in their correct groups after 7, 14, and 21 dpi, respectively. From the 9 to 10 control cases at 7, 14, and 21 dpi, all were correctly placed in their control group. However, one of the FHB cases was also placed in the control group after 7 and 14 dpi (67% correct) (Table 8). Twenty-one days after inoculation, all FHB cases on ‘Bjarne’ were correctly placed into the FHB group (Table 8). At all collection times, SNB on ‘Bjarne’ was correctly placed into the SNB group in 75% of the cases (Table 8). Powdery mildew cases were 100% correctly classified after 14 and 21 dpi, while one PM case was placed in the control group after 7 dpi (67% correct, Table 8). Cross validation showed a lower percentage of correct classification for all diseases at 7, 14, and 21 dpi, except for FHB at 7 and 14 dpi, SNB at 14 dpi, and PM at 7 and 14 dpi (Table 8).

**TABLE 7:** Summary of classification of fungal diseases on ‘Zebra’ with “disease” [control, Fusarium head blight (FHB), and Septoria nodorum blotch (SNB)] as the response and different VOCs as predictors.

	True group								
	7 days after inoculation^a^			14 days after inoculation			21 days after inoculation^b^		
Put into group	Control	FHB	SNB	Control	FHB	SNB	Control	FHB	SNB
Control	7	1	2	7	0	2	6	0	1
FHB	0	2	0	0	3	0	0	3	0
SNB	0	0	2	0	0	2	1	0	3
Total *N*	7	3	4	7	3	4	7	3	4
Correct *N*	7	2	2	7	3	2	6	3	3
Correctly classified (%)	100	67	50	100	100	50	86	100	75
Correctly classified (%) after cross validation	86	33	50	86	67	55	86	67	50

*The total number of samples, number of samples correctly classified, the proportion of correct classification, and the proportion of correct classification using cross validation are given in the table.*

*^a^After 7 and 14 days of inoculation, 3,5,5-trimethyl-2-hexene; sativene; germacrene D; and 2-heptadecanone were included as predictors.*

*^b^Germacrene D was strongly correlated with other predictors at 21 dpi and was therefore not included as predictor for that time point.*

**TABLE 8:** Summary of classification of fungal diseases on ‘Bjarne’ with “disease” [control, Fusarium head blight (FHB), Septoria nodorum blotch (SNB), and powdery mildew (PM)] as the response and different VOCs as the predictors.

	True group											
	7 days after inoculation				14 days after inoculation^b^				21 days after inoculation^c^			
Put into group	Control	FHB	SNB	PM	Control	FHB	SNB	PM	Control	FHB	SNB	PM
Control	10	1	1	1	9	1	1	0	9	0	1	0
FHB	0	2	0	0	0	2	0	0	0	3	0	0
SNB	0	0	3	0	0	0	3	0	0	0	3	0
PM	0	0	0	2	0	0	0	2	0	0	0	3
Total *N*	10	3	4	3	9	3	4	2	9	3	4	3
Correct *N*	10	2	3	2	9	2	3	2	9	3	3	3
Correctly classified (%)	100	67	75	67	100	67	75	100	100	100	75	100
Correctly classified (%) after cross validation	90	67	25	67	100	67	75	100	89	33	50	67

*^a^After 7 days of inoculation, 1-octen-3-ol; 3,5,5-trimethyl-2-hexene; 1-octanol; sativene; alpha-cedrene; germacrene D; and 2-heptadecanone were included as the predictors for classification of VOCs.*

*^b^After 14 days of inoculation, the predictors included 1-octen-3-ol; 3,5,5-trimethyl-2-hexene; sativene; 2-heptadecanone; and mullein.*

*^c^After 21 days of inoculation, the predictors included 1-octen-3-ol; 3,5,5-trimethyl-2-hexene; alpha-cedrene; germacrene D; α-patcholene; and mullein.*

When we combined all collection times for ‘Zebra’ and grouped the diseases based on the discriminant analysis, with the relevant VOCs as predictors and “control,” “FHB,” and “SNB” as response, 74% of the diseases cases were correctly classified. Ninety-one percent of the control cases were correctly classified, while 78% of FHB and 42% of SNB cases were correctly classified (Table 9). Cross validation showed the same results (Table 9). When we then combined all collection times for ‘Bjarne’ and grouped the diseases based on the discriminant analysis using different VOCs as predictors and “control,” “FHB,” “SNB,” and “PM” as responses, we classified 86% of all disease cases correctly. All of the control cases were correctly grouped into the control class (100%), while 78% of FHB, 75% of SNB, and 63% of PM cases were correctly classified (Table 9). Cross validation returned the same results except for SNB, when only 67% instead of 75% of the disease cases were correctly grouped (Table 9).

**TABLE 9:** Summary of classification of fungal diseases on ‘Zebra’ and ‘Bjarne,’ when data from 7, 14, and 21 days after inoculation was combined.

	True group						
	Zebra^a^			Bjarne^b^			
Put into group	Control	FHB	SNB	Control	FHB	SNB	PM
Control	19	2	7	28	2	3	3
FHB	0	7	0	0	7	0	0
SNB	2	0	5	0	0	9	0
PM	–	–		0	0	0	5
Total *N*	21	9	12	28	9	12	8
Correct *N*	19	7	5	28	7	9	5
Proportion of correct *N* (%)	91	78	42	100	78	75	63
Correctly classified (%) after cross validation	91	78	42	100	78	67	63

*Control, Fusarium head blight (FHB) and Septoria nodorum blotch (SNB) were the response factors, and different VOCs were the predictors for the classification of VOCs collected from ‘Zebra.’ Control, FHB, SNB, and powdery mildew (PM) were the response factors and different VOCs were the predictors for the classification of VOCs collected from ‘Bjarne.’*

*^a^3,5,5-Trimethyl-2-hexene; sativene; germacrene D, mellein; and 2-heptadecanone were set as the predictors for classification of VOCs collected from ‘Zebra.’*

*^b^1-Octen-3-ol; 3,5,5-trimethyl-2-hexene; alpha-cedrene; germacrene D; 2-(1-cyclopent-1-enyl-methylethethyl)cyclopentanone; 2-heptadecanone; and mellein were the predictors for classification of VOCs collected from ‘Bjarne.’*

Once we combined the data from both varieties and all collection times, using various VOCs as the predictors and “control,” “FHB,” “SNB,” and “PM” as the responses, the discriminant analysis placed 80% of the disease cases correctly into their respective disease group. Ninety-six percent of control, 78% of FHB, 54% of SNB, and 63% of PM cases were correctly grouped (Table 10). Cross validation returned similar results (Table 10). The classification of FHB and SNB based on VOCs produced by ‘Zebra’ (66.7 and 50%, respectively) (Table 7) and ‘Bjarne’ (67 and 75%, respectively) (Table 8) at 7 dpi was less accurate than the grouping based on these VOCs produced at 21 dpi [100 and 75% for ‘Zebra’ (Table 7) and 100 and 75% for ‘Bjarne’ (Table 8)].

**TABLE 10:** Summary of classification of fungal diseases on wheat (when data sets from ‘Bjarne’ and ‘Zebra’ 7, 14, and 21 days after inoculation were combined).

	True group			
	Zebra/Bjarne			
Put into group	Control (%)	FHB	SNB	PM
Control	47	4	11	3
FHB	0	14	0	0
PM	0	0	0	5
SNB	2	0	13	0
Total *N*	49	18	24	8
Correct *N*	47	14	13	5
Correctly classified (%)	96	78	54	63
Correctly classified after cross validation (%)	94	72	50	63

*“Disease” [including Fusarium head blight (FHB), Septoria nodorum blotch (SNB), and powdery mildew (PM)] was the response factor and 1-octen-3-ol; 3,5,5-trimethyl-2-hexene; sativene; germacrene D; α-patcholene; 2-(1-cyclopent-1-enyl-methylethethyl)cyclopentanone; mellein; and 2-heptadecanone were set as the predictors.*

#### The Relationship Between Volatile Organic Compound Emission and Disease Severity Across Wheat Varieties *in vivo*

**FIGURE 2:**
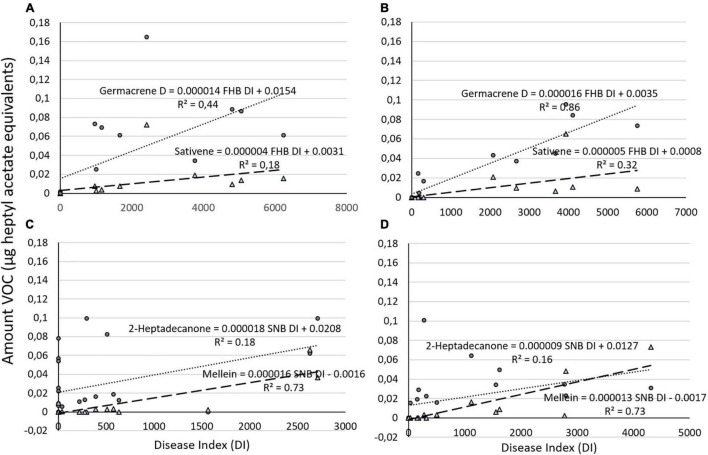
Graphical representation of amount of **(A)** two different volatile organic compounds (VOCs) (germacrene and sativene) regressed against Fusarium head blight (FHB) DI on ‘Zebra’ and **(B)** on ‘Bjarne’ and **(C)** of two different VOCs (2-heptadecanone and mellein) against Septoria nodorum blotch (SNB) DI on ‘Zebra’ and on **(D)** ‘Bjarne.’ The regression equations and the *R*^2^ values are placed next to the regression lines.

The regression analysis of emitted volatile compounds and their associated DI in both varieties showed generally a positive correlation. On ‘Zebra,’ FHB DI and germacrene D were significantly correlated and FHB DI explained 44% of the variation in germacrene D amounts (germacrene D_*Zebra*_ = 0.0154 + 0.000014 FHB DI, *R*^2^ = 0.44, *P* = 0.005), while FHB DI was not significantly correlated with sativene (sativene_*Zebra*_ = 0.0031 + 0.000004 FHB DI; *R*^2^ = 0.18, *P* = 0.078; Figure 2A). On ‘Bjarne,’ FHB DI was also significantly correlated with germacrene D and explained more than 85% of variation of this compound (germacrene d_*Bjarne*_ = 0.0035 + 0.000016 FHB DI, *R*^2^ = 0.86%, *P* < 0.001), while the production of sativene was also significantly correlated with FHB DI on ‘Bjarne’ (Figure 2B). However, FHB DI explained only 32% of variation in sativene (sativene_*Bjarne*_ = 0.0008 + 0.000005 FHB D, *R*^2^ = 0.32, *P* = 0.014; Figure 2B). Both on ‘Zebra’ and ‘Bjarne,’ the SNB DI was significantly correlated with mellein, while the SNB DI was only significantly correlated with 2-heptadecanone on ‘Zebra’ (*P* = 0.037) and not on ‘Bjarne’ (*P* = 0.057). The SNB DI explained 73% of variation in mellein production on both ‘Zebra’ and on ‘Bjarne’ (mellein_*Zebra*_ = −0.0016 + 0.000016 SNB DI, *R*^2^ = 0.73%, *P* < 0.001; Figure 2C and mellein_*Bjarne*_ = −0.0017 + 0.000013 SNB DI, *R*^2^ = 0.73%, *P* < 0.001; Figure 2D). On ‘Zebra,’ the SNB DI explained only 18% of variation in 2-heptadecanone (2-heptadecanone_*Zebra*_ = 0.0208 + 0.000018 SNB DI, *R*^2^ = 0.18%, *P* = 0.037; Figure 2C).

The analysis of variance of the regression analysis for the different VOCs and their respective DI showed that there was no significant difference between the VOC–DI relationships of the two varieties ‘Zebra’ and ‘Bjarne.’

**FIGURE 3:**
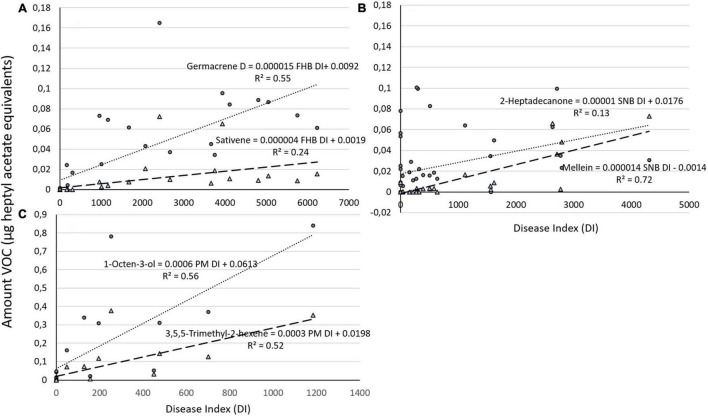
Graphical representation of amount of **(A)** two different VOCs (germacrene and sativene) regressed against Fusarium head blight (FHB) DI on both varieties (‘Zebra’ and ‘Bjarne’) combined and of **(B)** two different VOCs (2-heptadecanone and mellein) regressed against Septoria nodorum blotch (SNB) DI on both varieties combined. **(C)** Graphical representation of the amount of 1-octen-3-ol and 3,5,5-trimethyl-2-hexene regressed against powdery mildew (PM) DI on ‘Bjarne.’ The regression equations and the *R*^2^ value are placed next to the regression lines.

Once we combined the data for VOC production during the different times after inoculation for both varieties, the relationship between germacrene D and FHB DI and between sativene and FHB DI remained significant (*P* = 0.000 and *P* = 0.002, respectively). The DI for FHB explained 55% of variation in germacrene D (germacrene D = 0.0092 + 0.000015 FHB DI, *R*^2^ = 0.55) and 24% of sativene emission (sativene = 0.0019 + 0.000004 FHB DI, *R*^2^ = 0.24; Figure 3A). SNB DI for both varieties combined was significantly correlated with mellein and 2-heptadecanone (*P* = 0.001 and *P* = 0.013, respectively). The SNB DI still explained over 70% of variation in mellein emission in both ‘Zebra’ and ‘Bjarne’ combined (mullein = −0.0014 + 0.000014 SNB DI, *R*^2^ = 0.72) and 13% of 2-heptadecanone emission (2-heptadecanone = 0.0176 + 0.00001 SNB DI, *R*^2^ = 0.13; Figure 3B).

The relationships between the production of 1-octen-3-ol and the DI for PM and 3,5,5-trimethyl-2-hexene and PM DI on ‘Bjarne’ were both significant (*P* < 0.001 and *P* = 0.001, respectively). The PM DI explained over 50% of the variation in 1-octen-3-ol (1-octen-3-ol = 0.0613 + 0.00061 PM DI, *R*^2^ = 0.56) and 3,5,5-trimethyl-2-hexene emission (3,5,5-trimethyl-2-hexene = 0.0198 + 0.0003 PM DI, *R*^2^ = 0.52; Figure 3C).

## Discussion

Our studies showed that different fungal species produced different VOC profiles and could be grouped into their respective isolate and disease group based on these profiles with a medium to high accuracy (84.2% for both). Isolates from the same species produced very similar VOC profiles. Our results are in agreement with studies on fungal species isolated from indoor environments that showed that VOC profiles are species and sometimes even strain specific (Kuske et al., 2005). However, several studies showed that the VOC profile can change with the substrate and the environmental conditions at which the fungal isolates are grown (Kuske et al., 2005). We used only one media to cultivate the fungal isolates, and differences in the species-specific VOC profile would be expected if we had used different growth media under varying light, temperature, and humidity conditions. The same fungal isolate that is producing a certain VOC blend on PDA under controlled conditions could produce a completely different VOC blend when grown on plants out in the field.

In our study, some, but not all, VOCs produced by the pathogens in pure culture on PDA were also produced in significant amounts when the same fungal isolate had infected its wheat host. While 2-non-anone; 1-octen-3-ol; and 3,5,5-trimethyl-2-hexene were produced by *F. graminearum* isolate 201569 in pure culture, ‘Zebra’ and ‘Bjarne’ inoculated with this strain also emitted 3,5,5-trimethyl-2-hexene at 7, 14, and 21 dpi, but not always in amounts significantly different from the samples inoculated with other pathogens. At 14 and 21 dpi, 1-octen-3-ol was captured from FHB-inoculated plants, but it was only emitted in significantly different amounts by ‘Zebra’ at 14 dpi. Pure cultures of *P. nodorum* isolate 201254 emitted 2-non-anone; 1-octen-3-ol; 3,5,5-trimethyl-2-hexene, alpha-cedrene; germacrene D; α-patcholene; and mellein, while ‘Zebra’ and ‘Bjarne’ inoculated with the same strain emitted non-significant amounts of 3,5,5-trimethyl-2-hexene at 7 and 14 dpi and alpha-cedrene only at 7 and 21 dpi. However, significant amounts of mellein were emitted by SNB isolate 201254 in pure culture on PDA and by ‘Zebra’ and ‘Bjarne’ plants at 14 and 21 dpi with the same SNB isolate, suggesting that this compound is produced directly by the fungus under various growth conditions. Mellein and its derivatives are phytotoxic compounds directly produced by *P. nodorum via* the polyketide pathway (Huff and Hamilton, 1979; Devys et al., 1992). The compound 2-heptadecanone, which was emitted at 7 dpi of the two different wheat varieties inoculated with the SNB pathogen, but not by any of the fungal pathogens in pure culture, has been found in the essential oil of *Warionia saharae* (Znini et al., 2013) and other plant species, which might indicate that it is a general plant stress indicator rather than a specific pathogen indicator for SNB.

Germacrene D was emitted from ‘Zebra’ and ‘Bjarne’ plants inoculated with *F. graminearum* isolate 201569 at 7, 14, and 21 dpi. However, this strain did not consistently produce germacrene D in pure culture, and only *F. graminearum* isolate 140/80 did emit this compound in significant amounts. In addition, *F. poae* and *P. nodorum* isolates 201254, 201252, and 202586 produced germacrene D in significant amounts. This could indicate that this particular VOC is produced under certain growth conditions by isolates of different fungal species and might be not specific and reliable enough to serve as a pathogen-specific indicator in the field. Germacrene A and C, but not D, have been identified as metabolites in the terpene pathway of *Fusarium fujikuroi* (Burkhardt et al., 2016). Germacrene D is not unique to fungi, as it was also found in essential oils from several plant species (De Medeiros et al., 2016; Sampietro et al., 2016).

Sativene, on the other hand, was identified only from *F. graminearum*-inoculated plants but not from any of the *Fusarium* spp. isolates in pure culture. Flynn et al. (2019) reported that *F. graminearum* produces sativene when the fungus is grown in toxin induction medium, but not in non-induction medium. This suggests that sativene could be a pathogenicity factor in FHB, which might only be expressed in planta. As such, it would have the potential to serve as a pathogen-specific indicator for FHB in wheat if the amounts emitted would be sufficient for detection.

The VOC 2-(1-cyclopent-1-enyl-methylethyl)cyclopentanone associated with *F. graminearum* and *F. poae* in pure culture of our study has not been found in infected plants or emitted from fungi in any other study. Burdock (*Arctium lappa*) leaf essential oils contained 3,5,5-trimethyl-2-hexene, the compound found in ‘Bjarne’ inoculated with *B. graminis* f.sp. *tritici*, and was shown to inhibit *Escherichia coli* biofilms in earlier studies (Lou et al., 2013). This compound has not been described to be produced by fungi before. If it is commonly produced by wheat varieties infected with *B. graminis* f.sp. *tritici*, it could be a candidate as a pathogen-specific indicator for powdery mildew. The 2-non-anone is a methyl ketone found in many fruits and vegetables, wine, and beer. It was shown to be produced by *F. poae* on basic medium (van der Schaft et al., 1992), and it was contained in the VOC blend produced by the endophytic fungus *Muscodor albus* inhibiting *P. nodorum* (Strobel et al., 2001). However, it has not been associated with other wheat pathogenic fungi and has not been shown to be produced in pure culture by other *Fusarium* species or by *P. nodorum.* The compound 1-octen-3-ol was emitted in significant amounts by almost all isolates in pure culture, except for *F. avenaceum* 203587. It was also produced by ‘Bjarne’ infected with *B. graminis* f.sp. *tritici* in our study but interestingly not in ‘Bjarne’ or ‘Zebra’ when inoculated with FHB or SNB. This compound is known to be a major contributor to the characteristic mushroom aroma (Combet et al., 2006) and has been reported as a potential biomarker for *B. graminis* f.sp. *tritici* by Hamow et al. (2021). As *B. graminis* f.sp. *tritici* grows primarily on the surface of the plant, the characteristic mushroom smell might be emitted into the air by the superficial mycelium, while it might not be released from plants colonized by fungi growing in the interior of the plant. Both *F. graminearum* and *P. nodorum* grow inside their hosts, developing only pycnidia at the surface of the plant tissue that can emit any pathogen-specific VOC directly.

We demonstrated that VOC profiles of inoculated wheat plants were specific in the composition of the different VOCs emitted when infected with one pathogen at a time. There were differences in VOC profiles between wheat varieties infected with the same pathogen, but some compounds were emitted consistently from both varieties. Grouping the diseases based on a selection of the emitted VOCs over all sampling times leads to an overall accuracy of disease classification of 86% for ‘Bjarne’ and an overall accuracy of classification of 74% for ‘Zebra.’ Some compounds were emitted only during certain times after inoculation, but not during others, indicating that the timing of VOC collection is important for the actual detection of the pathogen.

The regression model based on the VOC profiles for both ‘Zebra’ and ‘Bjarne’ and DI of FHB, SNB, and PM showed that the relationship between VOC emission and disease intensity was variable, but significant. Increase in disease DI did not explain all the variations in specific VOC emission, supporting the idea that VOC blends or signatures are more useful in disease classification than single VOCs due to the many other factors influencing the emission of specific VOCs. However, the relationship between VOC emission and disease index could be useful to estimate the level of disease present based on the VOC concentration collected. There was no significant difference between the regression slope and the constant between the two wheat varieties, indicating that the relationship between VOC production and DI was robust across the two varieties tested. However, these models were developed based on plant inoculations under controlled greenhouse conditions, with only one single pathogen tested at a time, and cannot be expected to be transferrable to field conditions.

In our study, we collected VOC from wheat plants grown in the greenhouse and placed it in a closed container on a work bench in the laboratory for 24 h. We have not explored the possible effect of daytime, light, temperature or humidity, or additional stress factors, such as other pathogens, physical damage, drought, or nutrient deficiency. Under field conditions, a multitude of diseases can attack a plant simultaneously. This might lead to the simultaneous production of disease-specific VOCs we have observed in our study or to the production of other not-yet-determined VOC blends.

Our studies showed that different diseases and fungal pathogens can be classified into their respective disease group based on specific VOC profiles from wheat plants under controlled conditions. We also showed that the amounts of VOCs are correlated with the disease index of the infected plants. It is the first step toward exploiting the VOC-based chemical communication system of crop plants to direct site-specific control measures for more effective pathogen control in cereal production systems. In order to further explore the role of VOCs as biomarkers for wheat diseases, we would need to collect VOCs from wheat grown in the field and include earlier sampling times and sample from plants under simultaneous attack of different isolates and diseases.

## Data Availability Statement

The original contributions presented in the study are included in the article/supplementary material, further inquiries can be directed to the corresponding author.

## Author Contributions

AF designed and performed the experiments and wrote the manuscript. BA assisted with the experiments. HRN did the chemical analysis. All authors read the manuscript, gave editorial advice, and approved the submitted version.

## Conflict of Interest

The authors declare that the research was conducted in the absence of any commercial or financial relationships that could be construed as a potential conflict of interest.

## Publisher’s Note

All claims expressed in this article are solely those of the authors and do not necessarily represent those of their affiliated organizations, or those of the publisher, the editors and the reviewers. Any product that may be evaluated in this article, or claim that may be made by its manufacturer, is not guaranteed or endorsed by the publisher.

## Footnotes

1
http://www.amdis.net/
